# Transient paresthesia following polymyxin B loading dose in a critically ill patient: a case report

**DOI:** 10.1097/MS9.0000000000005278

**Published:** 2026-06-26

**Authors:** Albin Joshi, Safal Bogati, Sharad Khakurel, Santosh Acharya, Puskar Kunwor

**Affiliations:** a Department of Pharmacy, KIST Medical College and Teaching Hospital, Imadol, Lalitpur, Nepal; b Department of Internal Medicine, KIST Medical College and Teaching Hospital, Imadol, Lalitpur, Nepal; c Department of Critical Care, KIST Medical College and Teaching Hospital, Imadol, Lalitpur, Nepal; d Department of Clinical Pharmacy Services, Nepal Cancer Hospital and Research Center, Harisiddhi, Lalitpur, Nepal

**Keywords:** adverse drug reaction (ADR), antimicrobial toxicity, case report, critically ill, neurotoxicity, paresthesia, polymyxin, polymyxin B

## Abstract

**Background:**

Polymyxins, including colistin and polymyxin B, are considered salvage therapy to treat infections caused by multidrug-resistant gram-negative bacteria. Despite their importance, the clinical use of polymyxins is often constrained by their narrow therapeutic index, especially due to nephrotoxicity and neurotoxicity. Paresthesia associated with polymyxin B is often underreported, not fully understood, and only a few cases highlight this.

**Case presentation:**

We present a case of transient paresthesia occurring shortly after the administration of a loading dose of polymyxin B in a critically ill 33-year-old South Asian woman with intra-abdominal sepsis. Interestingly, the symptoms resolved spontaneously despite the continuation of the maintenance regimen.

**Conclusion:**

This case highlights the under-recognized neurotoxic potential of polymyxin B, particularly during loading doses, and underscores the importance of vigilance and individualized clinical judgment when balancing antimicrobial efficacy with tolerability.

## Introduction

Polymyxin B and colistin are critical therapeutic options for multidrug-resistant (MDR) gram-negative bacterial infections, including Enterobacteriaceae, *Pseudomonas aeruginosa*, and *Acinetobacter baumannii* species. While the nephrotoxic profile of polymyxins is well established, neurotoxicity is relatively underreported, often overshadowed by more urgent clinical parameters in the intensive care unit (ICU) setting. Neurological manifestations can range from mild paresthesia to serious complications such as neuromuscular blockade or respiratory depression. Sedation, altered consciousness, and concurrent use of other neuroactive agents frequently mask these symptoms in ICU patients, contributing to underdiagnosis^[^1^]^. Patients may experience a different set of symptoms, with varying severity. These symptoms include paresthesia (tingling or pricking sensation), diplopia (double vision), weakness, and respiratory failure. The toxic effects of polymyxins on both the kidneys and the nervous system are thought to be related to the dosage of the medication^[^2,3^]^.

HIGHLIGHTS

- Reports a case of transient paresthesia occurring shortly after a loading dose of polymyxin B in a critically ill patient.
- Neurological symptoms resolved spontaneously, despite the continuation of the maintenance dosing regimen.
- Findings underscore that neurotoxicity can manifest even in patients without classic risk factors, such as renal dysfunction or concurrent neurotoxic agents.
- Case supports the feasibility of continuing polymyxin B therapy under close monitoring when symptoms are mild and non-progressive.
- Highlights the need for clinical vigilance regarding polymyxin-associated neurotoxicity, particularly during loading doses in intensive care unit settings.

The FDA approves these agents for treating severe infections caused by MDR gram-negative organisms, particularly Enterobacteriaceae, *Pseudomonas aeruginosa*, and *Acinetobacter baumannii*^[^3^]^.

In this case report, we documented polymyxin B-associated neurotoxicity linked to the loading dose; however, the maintenance dose was continued under close vigilance, and the symptoms resolved. We believe that knowledge and expertise in identifying and managing potential drug-related neurotoxic symptoms are crucial to ensuring patient safety and providing optimal medication management. Such knowledge can help inform decisions on whether to discontinue or continue the medication based on the severity of reactions. In our case, we continued the maintenance dose with no further symptoms of paresthesia.

## Case report

A 33-year-old South Asian woman (weight: 50 kg) with portal hypertension (non-cirrhotic) and a history of multiple surgeries for recurrent common bile duct stones was admitted to the ICU at KIST Medical College Teaching Hospital on 4 June 2025, following hepaticojejunostomy. She had no prior comorbidities. Postoperatively, she developed signs of intra-abdominal infection. Initial bile cultures grew *Escherichia coli* and *Klebsiella oxytoca*, and she was started on meropenem and gentamicin. Despite culture-directed therapy, her clinical condition did not improve. Repeat cultures revealed *E. coli* and *Pseudomonas aeruginosa*.

On June 19, polymyxin B was initiated due to concern for MDR pathogens. Over 1 hour, a loading dose of 1 million IU in 300 mL of 5% dextrose water was administered. After an hour of completing the infusion, the patient reported diffuse tingling, numbness, and a burning sensation in the extremities, abdomen, and perianal area. Using a cotton swab to lightly touch the patient’s affected areas, sensory response was assessed on the extremities. The patient reported abnormal sensations of tingling and crawling as the swab touched her skin; the physician gave a score of 2/10 based on the visual analog scale (VAS). There was no associated weakness, respiratory difficulty, or alteration in sensorium. Her Glasgow Coma Scale score remained 15/15 throughout the period of symptom reporting.

The ICU intensivist consulted with the clinical pharmacy team regarding the possibility of drug-induced neurotoxicity caused by polymyxin B. Based on the clinician’s concern, the clinical pharmacist reviewed the medication to identify possible risk factors. Drug–drug interactions were checked among the prescribed medications; however, no significant interactions were identified that would contribute to neurotoxicity. Based on the literature reviewed, it was determined that neurotoxicity could be a possible adverse drug reaction (ADR) of polymyxin B. The pharmacist conducted a medication review and found that the patient had received the last dose of gentamicin 16 hours before the initiation of polymyxin B, so gentamicin was ruled out as a contributing medication for neurotoxicity. Other concurrent medications included pantoprazole, paracetamol, aspirin, metoclopramide, and total parenteral nutrition – none of which are commonly implicated in neurotoxicity. The patient’s oxygen saturation was maintained at 92% on room air. Electrolytes were within normal limits (Mg²^+^: 1.8 mg/dL, Ca²^+^8.2 mg/dL, K^+^: 3.8 mmol/L), and renal function was preserved (serum creatinine: 0.21 mg/dL; calculated creatinine clearance: 301 mL/min).

**Figure 1.**
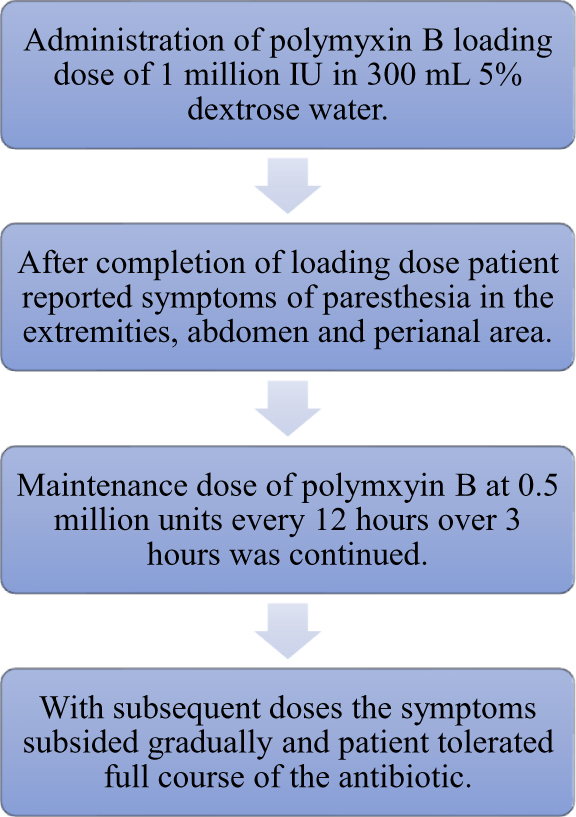
Flowchart of events leading to polymyxin B-associated paresthesia.

Given the mild nature of the symptoms and the lack of viable alternative antibiotics, a decision was made through a multidisciplinary team approach to proceed with the maintenance dose of polymyxin B at 0.5 million IU every 12 hours over 3 hours. The second dose elicited similar, but milder, paresthesia. On repeat assessment with the cotton swab, based on patient complaints, a VAS score of 1/10 was given. No further episodes occurred with subsequent doses, and the patient reported a feeling of light touch on the skin during assessment with a cotton swab and was scored 0/10 on the VAS. No adjunctive therapy was required, and the patient tolerated the full course of antibiotics, the timeline of events are displayed in Figure 1. Using the Naranjo scale, the adverse reaction was scored as “probable” with a total of 6.

The Naranjo Adverse Drug Reaction Probability Scale was used to evaluate the possibility of polymyxin B-induced paresthesia as a suspected ADR, as shown in Table 1. The total score on the Naranjo Scale was 6 and was classified as “probable.”

**Table 1:** Naranjo’s scale of ADR assessment.

Naranjo Adverse Drug Reaction Probability Scale	
Questions	Score
Are there previous conclusive reports of this reaction?	+1
Did the adverse event appear after the suspected drug was given?	+2
Did the adverse reaction improve when the drug was discontinued or a specific antagonist was given?	+1
Did the adverse reaction reappear upon re-administration?	+2
Are there alternative causes that could have caused the reaction?	−1
Did the reaction reappear when a placebo was given?	0
Was the drug detected in blood (or other fluids) at toxic concentrations?	0
Was the reaction more severe with increased dose, or less with reduced dose?	+1
Did the patient have a similar reaction to the same or similar drugs in the past?	0
Was the adverse event confirmed by any objective evidence?	0
Total score	6

## Discussion

Polymyxin B and colistimethate are the only available agents in the polymyxin family for clinical use due to their comparatively favorable safety profiles. They are among the last-line antibiotics for MDR gram-negative bacterial infections. Neurotoxicity with polymyxin B remains a relatively rare but clinically significant concern. While colistin has historically borne the brunt of this reputation, emerging data suggest polymyxin B may have a similar or even greater neurotoxic potential^[^4,5^]^ The literature describes various neurological manifestations, ranging from dizziness and ataxia to paresthesia and frank neuromuscular blockade.

Studies comparing these ADRs have shown a higher incidence of neurotoxicity associated with polymyxin B compared to colistin. However, in these studies, the drug was discontinued when symptoms of neurotoxicity were observed^[^4^]^. In a literature review, most neurotoxicity cases arose after the administration of the loading dose, albeit it was reported after several days of therapy initiation. According to them, the incidence of the adverse effect is estimated to 7.64%. Out of the 12 cases that attempted re-challenge (either with the same drug or dose reduction), 2 were successful^[^5^]^. In four case reports, symptoms of paresthesia associated with polymyxin B were resolved after switching to colistin, with tolerance to therapy maintained throughout the treatment duration^[^6^]^.

Our patient’s symptoms developed rapidly after the loading dose and resolved spontaneously with continued therapy, suggesting a dose-related, self-limiting adverse effect. Interestingly, she lacked classic risk factors such as renal dysfunction, neuromuscular disease, or concurrent neurotoxic agents. This highlights that even low-risk patients can develop neurotoxicity, especially following loading doses.

The International Consensus Guidelines for the Optimal Use of the Polymyxins recommend a loading dose of 2.0–2.5 mg/kg (equivalent to 20 000–25 000 IU/kg) and a maintenance dose of 1.25–1.5 mg/kg (equivalent to 12 500–15 000 IU/kg total body weight) every 12 hours^[^7^]^. Our patient received an appropriate loading dose (1 million IU ≈ 20 000 IU/kg), aligning with these guidelines. The pharmacodynamic rationale behind a loading dose is to rapidly achieve therapeutic concentrations, especially in critically ill patients. However, this may transiently raise serum levels beyond the neurotoxic threshold.

The pathophysiology of polymyxin-induced neurotoxicity remains incompletely understood but may involve interference with presynaptic acetylcholine release or direct neuronal membrane disruption^[^3^]^. Risk appears to increase with cumulative exposure, concomitant use of aminoglycosides, and female sex^[^8,9^]^ This case adds to the limited but growing body of literature supporting the feasibility of continuing therapy in the presence of mild, non-progressive symptoms.

Based on our case, for future practice, clinicians and other healthcare personnel need to be aware of the possible neurological ADRs associated with higher doses, especially in loading doses. Pharmacists can play an important role in identifying such ADRs and informing healthcare workers about further management. Here, there were not many risk factors present in the patient that could have led to symptoms severe enough to warrant treatment discontinuation. Nevertheless, in patients with risk factors such as renal dysfunction, concurrent neurotoxic agents, or neuromuscular disorders, careful consideration needs to be taken before the initiation of polymyxin B treatment due to its dose-dependent toxicity.

## Conclusion

This case highlights a clinically relevant but underappreciated adverse effect of polymyxin B – transient paresthesia following a loading dose in a critically ill patient. In the absence of progression or additional neurological findings, continued therapy with close observation may be appropriate. Given the rising use of polymyxins in the era of antimicrobial resistance, clinicians must remain vigilant for such adverse effects and tailor therapy based on individual risk–benefit considerations.

## Data Availability

All data generated or analyzed during this study are included in this published article.

## Research registration unique identifying number (UIN)

Not applicable – Registration is not required for case reports.
